# Primary, atrial secondary, and ventricular secondary mitral regurgitation in the general population

**DOI:** 10.1007/s00392-026-03046-7

**Published:** 2026-10-27

**Authors:** Laura Keil, Alexander Hapfelmeier, Jan-Per Wenzel, Julius Nikorowitsch, Daniel Kalbacher, Hermann Reichenspurner, Stefan Blankenberg, Evaldas Girdauskas

**Affiliations:** 1 Department of General and Interventional Cardiology, University Heart and Vascular Center Hamburg, Hamburg, Germany; 2 German Center for Cardiovascular Research (DZHK), Partner Site Hamburg/Kiel/Luebeck, Hamburg, Germany; 3 Institute of AI and Informatics in Medicine, TUM School of Medicine and Health, Technical University of Munich, Munich, Germany; 4 Institute of General Practice and Health Services Research, TUM School of Medicine and Health, Technical University of Munich, Munich, Germany; 5 Department of General and Interventional Cardiology, University Medical Center Luebeck, Luebeck, Germany; 6 Department of General and Interventional Cardiology, Albertinen Hospital, Hamburg, Germany; 7 Department of Cardiovascular Surgery, University Heart and Vascular Center Hamburg, Hamburg, Germany; 8 Epidemiological Study Center, Hamburg, Germany

**Keywords:** Primary mitral regurgitation, Secondary mitral regurgitation, Ventricular SMR, Atrial SMR, Echocardiography, Hamburg City Health Study

## Abstract

**Background:**

While primary mitral valve regurgitation (PMR) results from structural abnormalities of the mitral valve leaflets, secondary mitral valve regurgitation (SMR) arises from either mitral annular dilatation (atrial type, aSMR) or left ventricle remodelling (ventricular type, vSMR) in the absence of intrinsic leaflet disease. Although SMR is considered the most common cause of clinically relevant mitral regurgitation (MR), population-based data on its prevalence, subtype distribution, and clinical profiles are lacking. We aimed to determine the prevalence of PMR and SMR and to characterize PMR, aSMR, and vSMR in the general population.

**Methods:**

We analyzed the first 10,000 participants of the population-based Hamburg City Health Study enrolled 2016–2019; 8543 echocardiographic examinations were available. Mitral valve morphology and function were independently assessed by two investigators. PMR was defined by the presence of intrinsic leaflet pathology, whereas SMR was classified as ventricular when left ventricular ejection fraction was less than 50% or less than 55% in the presence of geometric distortion (tenting area ≥ 150 mm^2^ or coaptation depth ≥ 10 mm) and cases with left ventricular ejection fraction equal to or above 50% were classified as aSMR.

**Results:**

A total of 216 participants had moderate or severe MR (2.53% of the total cohort, 95% confidence interval (CI): 2.20%–2.89%, see Fig. 3). Of these, 148 were classified as SMR (68.5%, 95% CI: 61.8%–74.7%), the remaining were mainly of primary etiology (31.5%, 95% CI: 25.3%–38.2%). Among SMR cases, aSMR was predominant (84.5%, 95% CI: 77.5%–89.9%), whereas vSMR accounted for 15.5% (95% CI: 10.1%–22.4%). Compared with aSMR, individuals with vSMR were older, more symptomatic, and more frequently had atrial fibrillation and hypertension. Echocardiographic parameters indicated more advanced cardiac remodelling and dysfunction in vSMR. Compared with SMR, participants with PMR had greater MR severity, while age and the proportion of women were numerically higher without reaching statistical significance. During a median follow-up of 4.56 years, 10 participants had died, with all-cause mortality being significantly higher among participants with PMR than among those with aSMR.

**Conclusion:**

In this population-based cohort, SMR accounted for two-thirds of relevant MR, with aSMR being the predominant subtype. MR severity was higher in PMR as compared to SMR. Distinct echocardiographic and clinical profiles suggest more advanced disease in vSMR. Multivariate analysis revealed that atrial fibrillation was significantly more present in the vSMR cohort. These findings highlight the need for refined SMR subclassification and longitudinal studies to define prognostic implications and clinically meaningful definitions. All-cause mortality was highest in participants with PMR than in those with aSMR.

**Graphical Abstract:**

**Figure:**
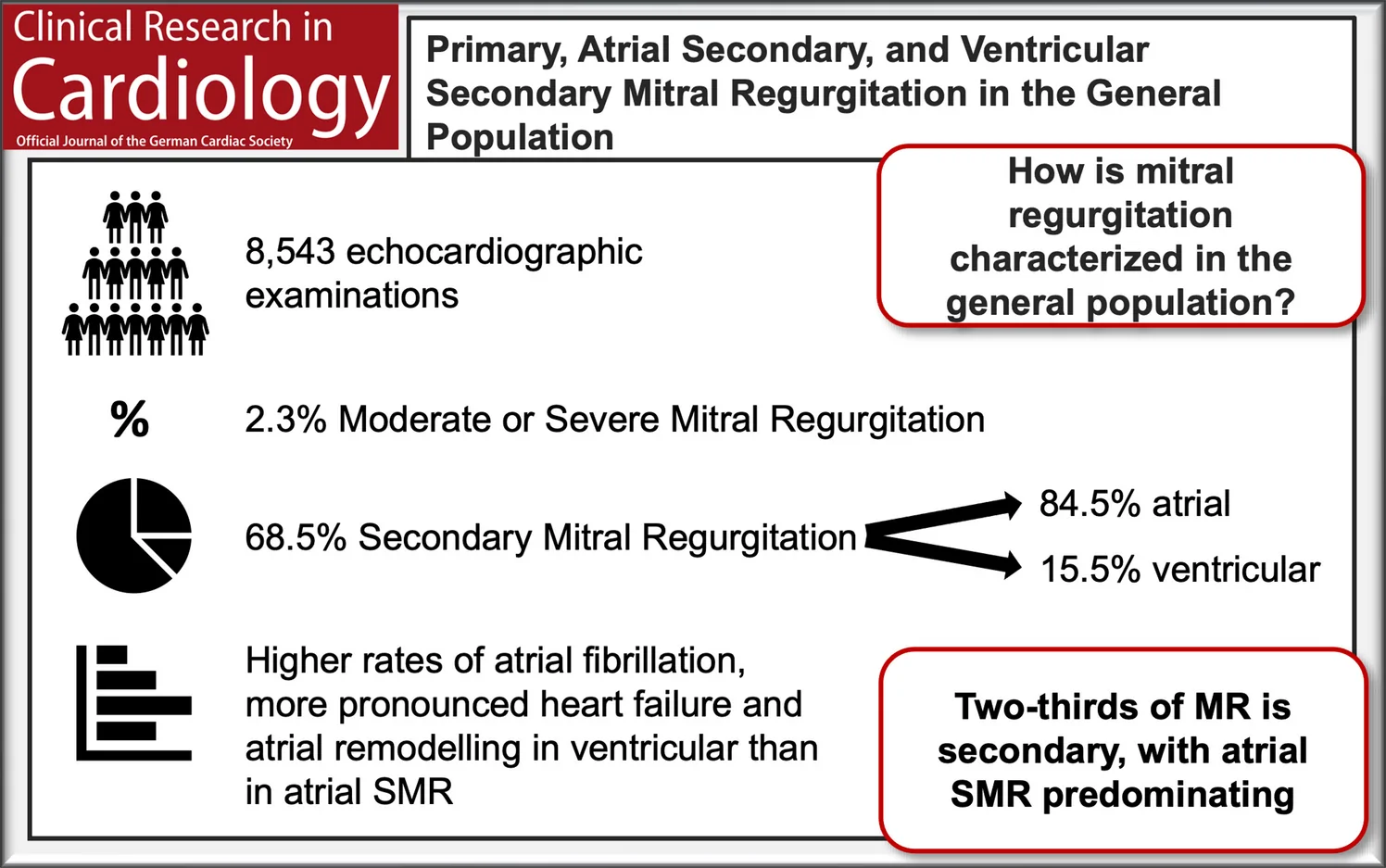

## Introduction

**Fig. 1:**
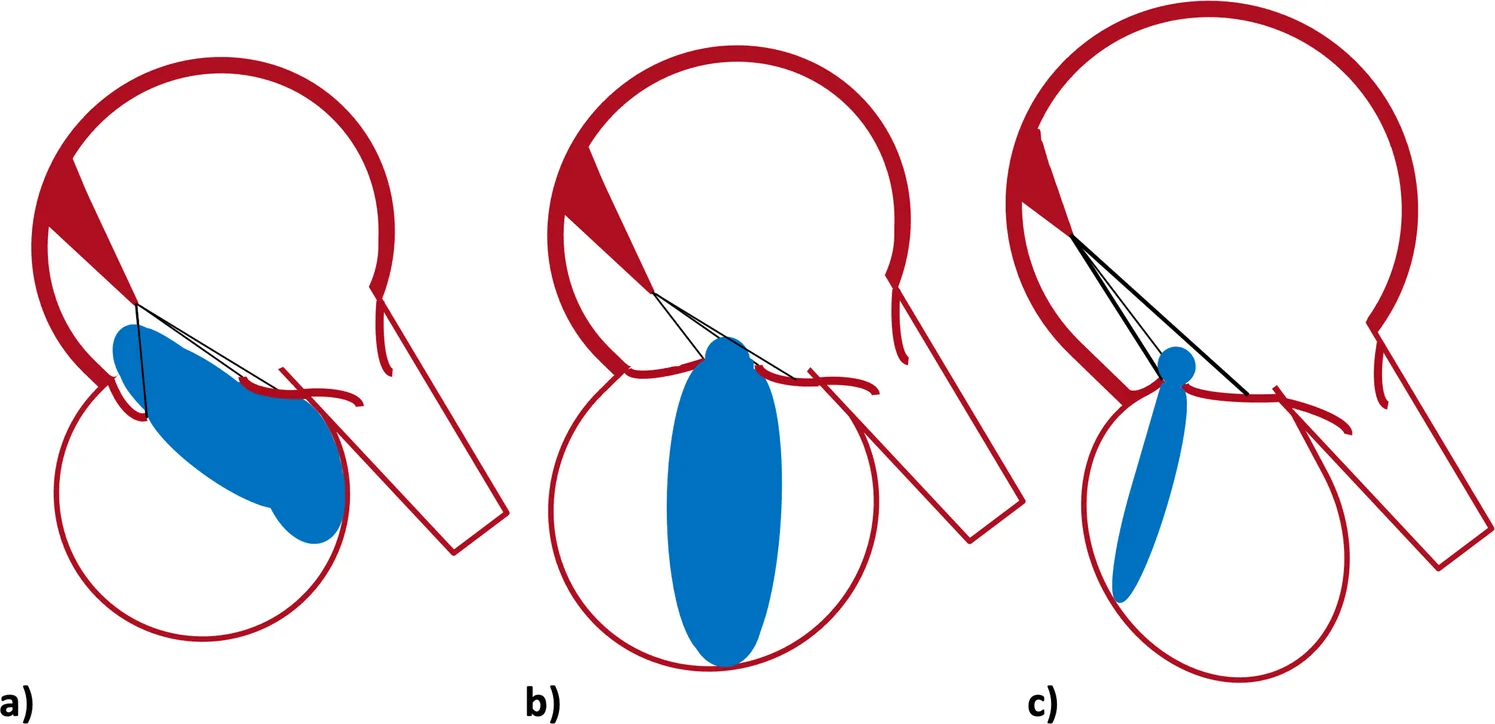
Comparison of primary mitral valve regurgitation **a**, atrial secondary mitral valve regurgitation **b**, and ventricular secondary mitral valve regurgitation **c**

**Fig. 2:**
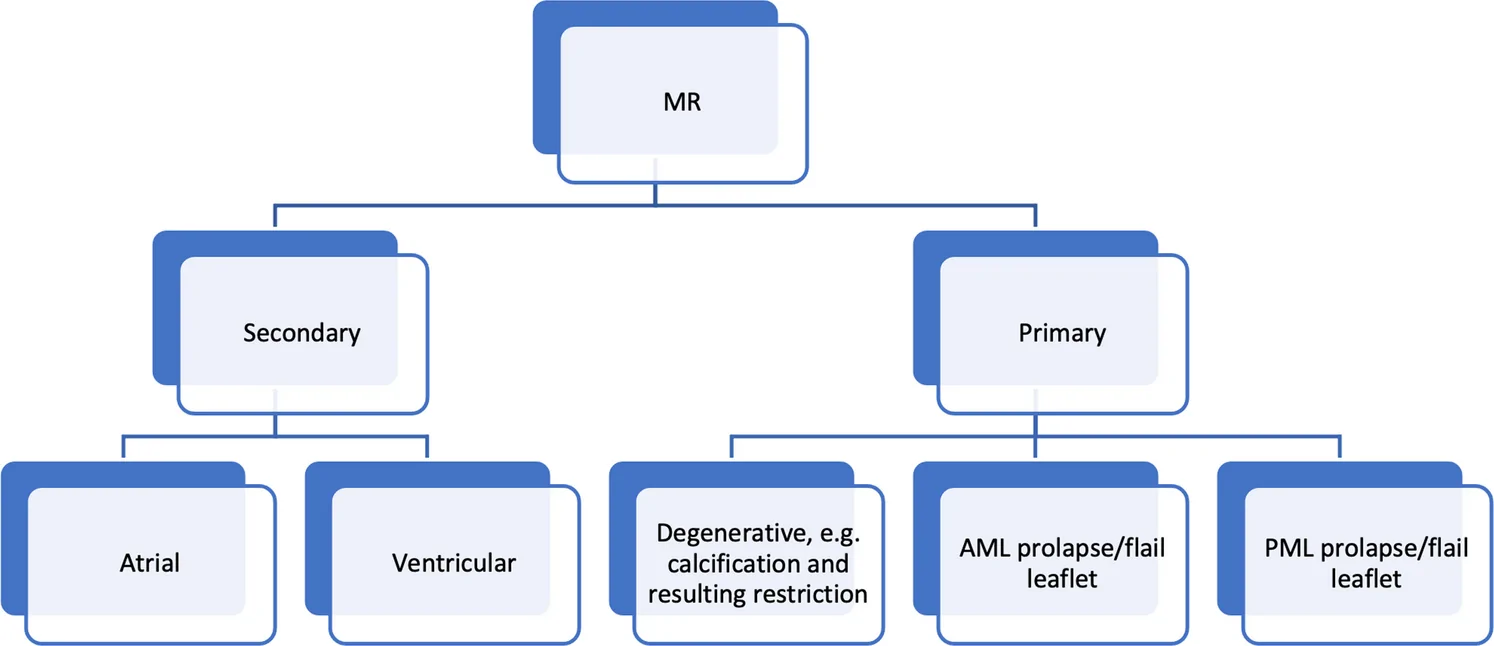
Classification of mitral valve regurgitation

Among the elderly population, mitral valve regurgitation (MR) is the second most common valve pathology [1]. Secondary mitral regurgitation (SMR), the predominant form of MR, is defined as MR occurring in the presence of structurally normal mitral valve leaflets [2, 3]. The primary pathophysiological mechanisms underlying SMR include (1) dilatation of the mitral annulus (atrial type) or (2) left ventricular remodelling (ventricular type), in the absence of intrinsic leaflet pathology (Fig. 1). Annular or atrial MR (aSMR) is typically observed in patients with long-standing atrial fibrillation or heart failure with preserved ejection fraction (HFpEF) and is associated with pronounced left atrial dilatation [4]. In contrast, ventricular SMR (vSMR) arises as a consequence of underlying left ventricular (LV) disease of either ischemic or non-ischemic etiology, including dilated cardiomyopathy (DCM) [4, 5]. In vSMR, the principal mechanism leading to MR is geometric distortion of the left ventricle, resulting in papillary muscle displacement and leaflet tethering [4]. In contrast, primary MR (PMR) results from intrinsic structural abnormalities of the mitral valve leaflets, chordae tendineae, or papillary muscles rather than from annular or ventricular remodeling (Fig. 1) [6, 7]. For a schematic classification of MR phenotypes, see Fig. 2.

Although SMR has been reported to be the most common cause of clinically relevant MR, population-based data on its prevalence—and on the relative distribution of its two main phenotypes (atrial vs. ventricular SMR)—remain scarce. Furthermore, robust data on the risk profiles of patients with atrial and ventricular SMR, their long-term prognosis, and comparisons with PMR remain limited [2]. Consequently, there is an urgent need for an updated, population-based evaluation of PMR and SMR prevalence, phenotypic distribution, and associated clinical outcomes.

## Methods

### Study population

The study included the first 10,000 participants of the prospective, population-based Hamburg City Health Study (HCHS), enrolled between 2016 and 2019. All residents of Hamburg aged 45 to 74 years were eligible for participation. Individuals were randomly selected and invited to undergo a baseline examination at the Study Center Hamburg-Eppendorf. This visit comprised a comprehensive transthoracic echocardiographic assessment (TTE), standardized questionnaires, and laboratory testing. A detailed description of the study design, participation selection, and data acquisition has been published previously [8].

In brief, TTE was performed by trained medical personnel using a Siemens Acuson SC200 Prime ultrasound system (Siemens Healthineers, Erlangen, Germany), following predefined standard operating procedures (SOPs) and in accordance with current guidelines of the American Society of Echocardiography (ASE) and the European Association of Cardiovascular Imaging (EACVI) [9].

### Ethical considerations

The study protocol was reviewed and approved by the local ethics committee of the Hamburg Chamber of Physicians (Landesärztekammer Hamburg, PV5131). The HCHS is registered at ClinicalTrial.gov (NCT03934957). All study procedures were conducted in accordance with the principles of Good Epidemiological Practice and the Declaration of Helsinki. Written informed consent was obtained from all participants prior to enrollment. A detailed description of the study design and ethical framework has been published elsewhere [8].

### Mitral valve assessment

Mitral valve morphology and function were assessed independently by two investigators. Standard transthoracic echocardiographic views, including the apical four-chamber (4CH), two-chamber (2CH), and three-chamber (3CH) views, as well as parasternal long axis (PLAX) and parasternal short axis (SAX) views, were systematically evaluated and graded by two independent, trained cardiologists.

The following parameters were assessed qualitatively: severity of mitral regurgitation; overall image quality; leaflet mobility (normal, restrictive anterior mitral leaflet (AML), restrictive posterior leaflet (PML), prolapse or flail of AML, prolapse or flail of PML); extent and distribution of valvular calcification (mild, moderate, severe; isolated AML or PML involvement); and regurgitation jet characteristics (central, eccentric anterior, eccentric posterior, or multiple jets).

Quantitative measurements were performed manually and independently by both trained cardiologists and included mitral annular diameter (PLAX, enddiastolic), tenting area (PLAX), coaptation depth (4CH or PLAX), AML angle (4CH or PLAX), PML angle (4CH or PLAX), left ventricular end-diastolic and end-systolic diameters (PLAX), and the left ventricular sphericity index (4CH).

PMR was defined by the presence of intrinsic leaflet pathology (i.e., prolapse, flail leaflet, or severe calcification or thickening resulting in restrictive leaflet motion). SMR was classified as ventricular in the presence of a left ventricular ejection fraction < 50% or < 55% in combination of geometric distortion, defined as a tenting area ≥ 150 mm^2^ or a coaptation depth ≥ 10 mm. In contrast, cases with preserved (left ventricular ejection fraction ≥ 50%) were classified as aSMR.

This classification was chosen to provide a pragmatic and clinically relevant definition, consistent with previously published studies [4, 6].

### Follow-up

Between October 2022 and April 2023, follow-up data on vital status and incident morbidity were collected from HCHS participants. Vital status was obtained from relatives and the population registry, while incident diseases and dates of diagnosis were assessed by questionnaire. Selected self-reported endpoints were subsequently adjudicated using medical records and information from treating physicians.

### Statistical analysis

Descriptive statistics are presented as absolute and relative frequencies (percentages) for categorical variables and as medians with interquartile range (IQRs) for continuous variables. Group comparisons were performed using two-sided Mann–Whitney *U* or Kruskall–Wallis tests for continuous variables and chi-squared tests for categorical variables. Two-sided Clopper–Pearson confidence intervals were calculated for inference on relative frequencies.

Univariable and prespecified multivariable binary logistic regression models were used to estimate odds ratios (ORs) with corresponding two-sided 95% confidence intervals (95% CIs) as measures of association between the investigated variables and the binary outcome of interest (vSMR vs. aSMR and PMR vs. SMR). To ensure stable and consistent effect estimation, multivariable models were restricted to a maximum of five regression coefficients [10]. Median follow-up was estimated by the reverse Kaplan–Meier method censoring death. The risks of death, atrial fibrillation and heart failure were estimated by cumulative incidence functions in this setting of competing risks. Gray tests were used for respective group comparisons implementing a closed testing procedure to control the overall type I error. There were no multivariable time-to-event analyses due to the low number of observed events.

All statistical hypothesis testing was exploratory in nature and conducted at a two-sided significance level of 5%. Statistical analyses were performed using R version 4.6.1 (R Foundation for Statistical Computing, Vienna, Austria).

## Results

### Overall

Of the 10,000 participants included in this study, 8543 echocardiographic examinations were available for analysis.

In this population-based cohort, the median age was 69.0 years (61.0–63.0), and nearly 50% of participants were female. Overall, median left ventricular ejection fraction (58.4%) was within the normal range, as were median left atrial biplane volume index (31.5 ml/m^2^) and left ventricular end-diastolic diameter (48.6 mm). Median NT-proBNP (N-terminal pro-B-type natriuretic peptide) levels were only mildly elevated (163.0 ng/l). Most participants were only mildly symptomatic, with 87.0% classified as NYHA functional class I. Cardiovascular comorbidities, including heart failure (21.3%), atrial fibrillation (19.2%), coronary artery disease (9.5%), and diabetes mellitus (5.5%), were relatively infrequent, whereas hypertension was highly prevalent (73.5%).

**Fig. 3:**
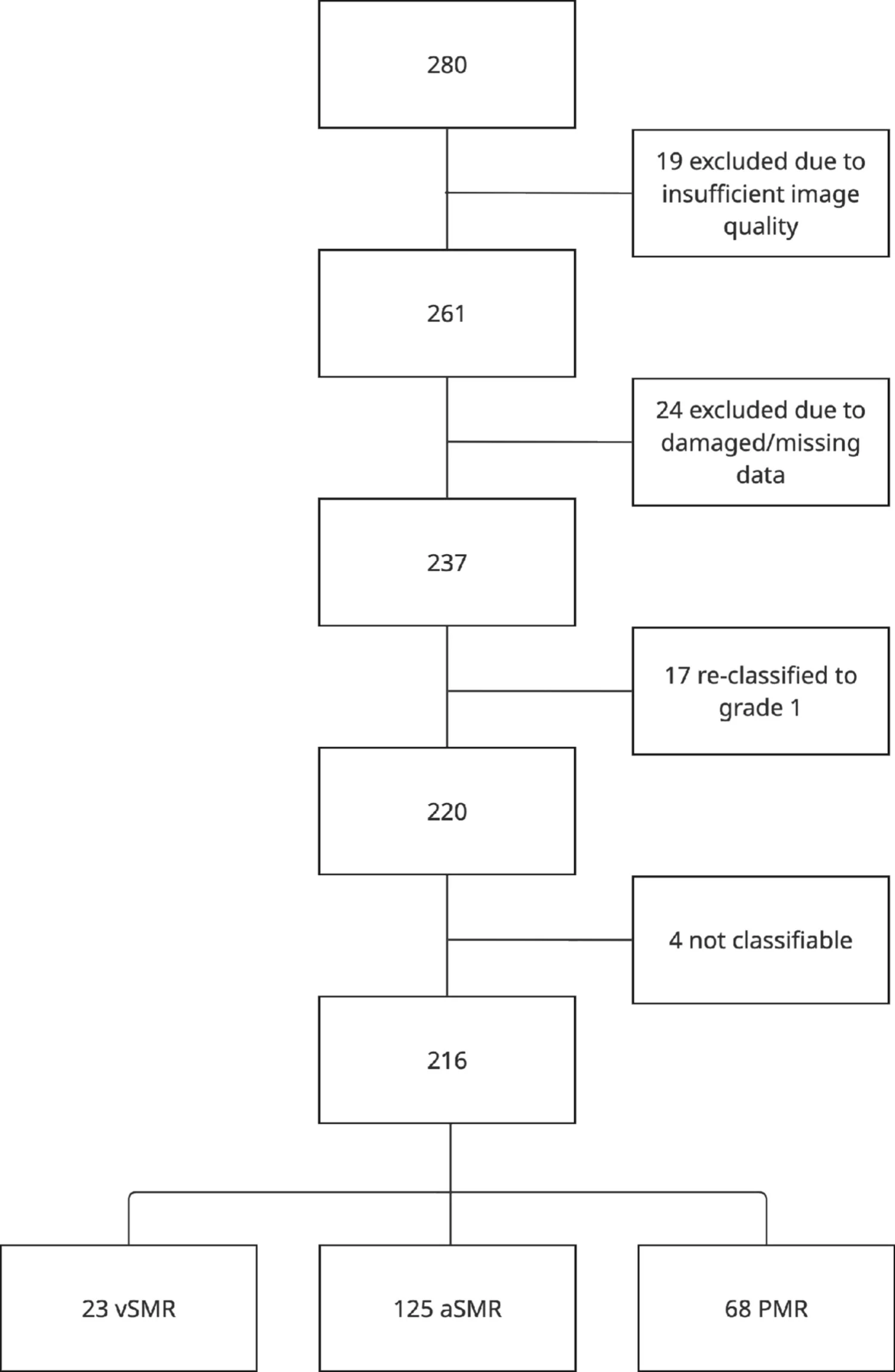
Patient flow chart

Within this cohort, 280 individuals were identified as having moderate or severe MR. After exclusion of cases with insufficient image quality, missing data, or reclassification to grade 1 by the second investigator, 216 cases remained, corresponding to a prevalence of 2.53%. Of these, 148 cases were classified as SMR (68.5%) and 68 as PMR (31.5%). According to the predefined classification, 23 cases were categorized as vSMR and 125 as aSMR (Fig. 3).

### aSMR vs. vSMR

With respect to demographic characteristics, no statistically significant differences were observed between individuals with aSMR and vSMR regarding sex, body mass index, or major cardiovascular risk factors, including smoking status, hypertension, and diabetes mellitus (Table 1). However, participants with vSMR tended to be older (*p* = 0.059) and were less frequently female (*p* = 0.078) compared with those with aSMR. Notably, only 16.1% of individuals in the aSMR group had atrial fibrillation.

**Table 1:** Demographics, comorbidities and laboratory data. Descriptive statistics are median (IQR) or absolute frequency (percentage). Numbers of missing values are reported in brackets after each variable, where applicable

	PMR	aSMR	vSMR	Total	*p*-value	*p*-value	*p*-value
	*N* = 68	*N* = 125	*N* = 23	*N* = 216 (100%)	PMR/aSMR/vSMR	aSMR/vSMR	PMR/SMR
***Demographics***							
Age, years	70.0 (64.0–74.0)	68.0 (60.0–72.0)	70.0 (64.0–74.5)	69.0 (61.0–63.0)	0.042	0.059	0.083
Female	38 (55.9%)	63 (50.4%)	7 (30.4%)	108 (49.3%)	0.107	0.078	0.241
BMI, kg/m^2^(7/7/1/15)	25.1 (22.5–28.3)	25.6 (23.3–28.2)	25.7 (23.5–27.8)	25.5 (23.1–28.3)	0.932	0.823	0.774
Current smoker (0/1/0/1)	6 (8.8%)	6 (4.8%)	3 (13.0%)	15 (7.0%)	0.482	0.207	0.753
History of smoking	30 (44.1%)	61 (49.2%)	8 (34.8%)	99 (46.0%)			
Never	32 (47.1%)	57 (46.0%)	12 (52.2%)	101 (47.0%)			
Sport in summer in hours/week (17/15/5/37)	2.5	2.0	1.5	2.0	0.110	0.151	0.130
Sport in winter in hours/week (20/17/5/42)	2.3	2.0	1.3	2.0	0.065	0.100	0.101
Myocardial infarction father (28/53/10/91)	9 (22.5%)	14 (19.4%)	3 (23.1%)	26 (20.8%)	0.909	0.763	0.748
Myocardial infarction mother (21/40/8/69)	4 (8.5%)	11 (12.9%)	0 (0.0%)	15 (10.2%)	0.280	0.140	0.642
Stroke father (31/51/9/91)	11 (29.7%)	8 (10.8%)	0 (0.0%)	19 (15.2%)	0.008	0.197	0.003
Stroke mother (23/41/7/71)	11 (24.4%)	18 (21.4%)	2 (12.5%)	31 (21.4%)	0.606	0.413	0.546
**Comorbidities**							
Hypertension (2/3/0/5)	49 (74.2%)	88 (72.1%)	18 (78.3%)	155 (73.5%)	0.817	0.543	0.862
Diabetes (6/9/0/15)	5 (8.1%)	6 (5.2%)	0 (0.0%)	11 (5.5%)	0340	0.265	
Coronary artery disease (13/7/6/26)	9 (16.4%)	5 (4.2%)	4 (23.5%)	18 (9.5%)	0.005	0.003	0.010
Atrial fibrillation (1/1/0/2)	14 (20.9%)	20 (16.1%)	7 (30.4%)	41 (19.2%)	0.252	0.104	0.663
Heart failure (14/17/2/33)	15 (27.8%)	13 (12.0%)	11 (52.4%)	39 (21.3%)	< 0.001	< 0.001	0.167
NYHA (15/12/5/32)							
Grade I	40 (75.5%)	104 (92.0%)	16 (88.9%)	160 (87.0%)	0.043	0.655	0.008
Grade II	12 (22.6%)	9 (8.0%)	2 (11.1%)	23 (12.5%)			
Grade III	1 (1.9%)	0 (0.0%)	0 (0.0%)	1 (0.5%)			
ESC SCORE2 (8/14/1/23)	9.9 (6.9–13.6)	8.2 (4.9–12.6)	12.5 (7.6–14.5)	9.6 (5.8–13.4)	0.075	0.052	0.261
**Laboratories**							
LDL, mg/dl	40.0 (24.5–69)	52.5 (24.0–85.0)	43.0 (16.5–75.5)	47.5 (23.8–81.3)	0261	0.235	0.262
NT-proBNP, ng/l (6/1/0/7)	167.5 (103.0–489.5)	143.5 (85.3–368.0)	445.0 (118.5–960.5)	163.0 (87.0–467.0)	0.092	0.046	0.405

*BMI* body mass index, *NYHA* New York Heart Association, *ESC SCORE2* European Society for Cardiology Systematic COronary Risk Evaluation, *LDL* low-density lipoprotein, *NT-proBNP* N-terminal pro-brain natriuretic peptide

Regarding comorbidities, participants with vSMR more frequently had coronary artery disease (*p* = 0.003) and self-reported heart failure (*p* < 0.001) compared with those with aSMR. In addition, individuals in the vSMR group more often reported advanced dyspnea and atrial fibrillation, which was paralleled by higher circulating NT-proBNP levels (*p* = 0.046).

**Fig. 4:**
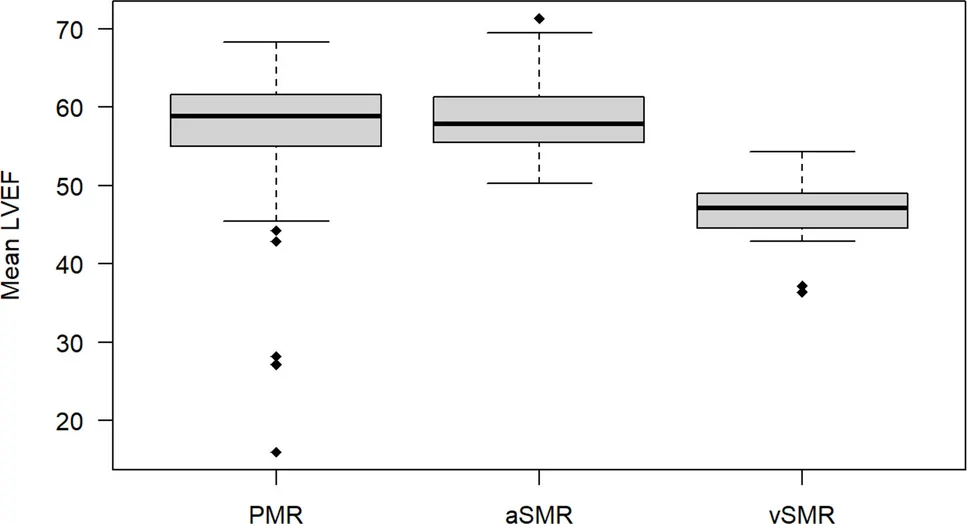
Boxplots comparing the distribution of left ventricular ejection fraction (PMR = primary MR, aSMR = atrial SMR, vSMR = ventricular SMR)

When comparing echocardiographic parameters, there was no statistically significant difference in MR severity between the subgroups. As expected by definition, participants with vSMR exhibited a more severely impaired left ventricular ejection fraction (LVEF) compared with those with aSMR (Table 2, Fig. 4). In addition, individuals with vSMR demonstrated a higher E/e′ ratio and a lower left atrial ejection fraction.

**Table 2:** Echocardiographic data. Descriptive statistics are median (IQR). Numbers of missing values are reported in brackets after each variable, where applicable

	**PMR**	**aSMR**	**vSMR**	**Total**	***p*-value**	***p*-value**	***p*-value**
	*N* = 68	*N* = 125	*N* = 23	*N* = 216 (100%)	PMR/aSMR/vSMR	aSMR/vSMR	PMR/ SMR
**General echocardiographic data**							
LVEF bp 2D, % (22/32/6/60)	58.4 (55.7–62.0)	57.9 (55.8–60.8)	47.4 (44.3–48.9)	57.6 (54.6–60.8)	< 0.001	< 0.001	0.068
LV stroke index bp (23/33/6/62)	34.7 (28.7–38.8)	33.3 (28.5–39.5)	24.9 (19.1–30.2)	32.8 (27.9–38.7)	< 0.001	< 0.001	0.303
IVSd; mm (7/7/1/15)	10.2 (8.8–11.5)	9.9 (9.0–11.0)	10.9 (9.3–11.6)	10.0 (8.9–11.4)	0.325	0.144	0.761
LVEDD; mm (12/12/1/25)	47.8 (45.6–53.4)	48.9 (45.7–43.2)	49.6 (45.2–54.7)	48.6 (45.7–53.6)	0.880	0.642	0.780
E′ Lateral mitral annulus, cm/s (8/4/1/13)	0.1 (0.1–0.1)	0.1 (0.1–0.1)	0.1 (0.1–0.1)	0.1 (0.1–0.1)	0.144	0.904	0.050
E′ Medial mitral annulus’, cm/s (8/10/3/21)	0.1 (0.1–0.1)	0.1 (0.1–0.1)	0.1 (0.1–0.1)	0.1 (0.1–0.1)	0.612	0.916	0.326
E/e′ mean ratio (12/15/4/31)	8.2 (7.1–11.8)	7.6 (6.4–9.1)	8.5 (7.2–9.6)	8.0 (6.6–9.6)	0.027	0.131	0.024
E/A ratio (15/12/6/33)	1.0 (0.7–1.2)	1.0 (0.8–1.2)	0.9 (0.7–1.1)	1.0 (0.8–1.2)	0.153	0.095	0.293
TAPSE, mm (17/35/7/59)	2.3 (2.0–2.6)	2.4 (2.0–2.7)	2.2 (1.7–2.5)	2.4 (2.0–2.7)	0.364	0.154	0.870
LADV bp; ml (22/29/5/56)	32.3 (25.0–60.7)	30.5 (21.7–47.5)	45.6 (23.2–74.3)	31.5 (22.8–52.5)	0.158	0.109	0.234
LAEF bp, % (22/31/6/59)	39.7 (27.5–46.8)	46.0 (38.7–52.9)	32.6 (19.8–39.3)	43.9 (33.7–51.9)	< 0.001	0.004	0.022
MV PGmax, mmHg (55/96/17/168)	4.0 (2.2–6.6)	2.8 (2.3–3.5)	3.5 (3.0–4.2)	2.9 (2.3–4.2)	0.223	0.101	0.313
LAS global peak all (31/50/13/94)	33.1 (25.3–43.9)	31.5 (23.3–40.5)	23.4 (15.0–32.4)	31.2 (23.3–41.9)	0.194	0.134	0.284
LASV_BSA bp (26/36/5/67)	32.8 (25.5–47.0)	31.2 (23.5–42.5)	33.2 (25.8–44.4)	31.3 (24.4–43.7)	0.572	0.459	0.458
**Mitral valve characterisation**							
Mitral valve regurgitation grading					0.018	0.959	0.003
Moderate	26 (38.2%)	78 (62.4%)	13 (56.5%)	117 (54.2%)			
Moderate to severe	1 (1.5%)	0 (0.0%)	0 (0.0%)	1 (0.5%)			
Severe	41 (60.3%)	47 (37.6%)	10 (43.5%)	98 (45.4%)			
MV annulus, mm	25.6 (22.3–30.5)	24.8 (21.8–29.8)	29.2 (23.1–32.6)	25.2 (21.9–30.6)	0.126	0.043	0.601
Tenting area, cm^2^	86.2 (63.6–126.7	107.3 (78.6–135.2)	114.2 (85,5–160.4)	105.9 (76.9–136.2)	0.029	0.227	0.015
Coaptation depth, mm (1/0/0/1)	6.8 (5.6–7.9)	7.2 (6.3–8.3)	6.3 (5.2–7.9)	7.0 (6.0–8.1)	0.09	0.085	0.157
Angle anterior leaflet, mm	35.0 (24.0–44.0)	36.0 (29.0–43.0)	29.0 (24.5–35.5)	35.0 (27.0–42.3)	0.027	0.006	0.520
Angle posterior leafleft,mm	38.0 (28.8–46.3)	43.0 (36.0–52.0)	45.0 (34.0–48.0)	42.0 (33.0–50.3)	0.02	0.471	0.007
Sphericity index (2/2/0/4)	0.6 (0.6–0.7)	0.6 (0.6–0.7)	0.6 (0.6–0.7)	0.6 (0.6–0.7)	0.118	0.045	0.801

*LVEF bp *left ventricular ejection fraction biplane, *IVSd* interventricular septum thickness at end-diastole, *LVEDD *left ventricular enddiastolic volume, *TAPSE *tricuspid annular plane systolic excursion, *LADV *left atrial diastolic volume, *LAEF *left atrial ejection fraction, *MV *mitral valve, *LAS *left atrial strain, *LASV_BSA *left atrial volume indexed to body surface area

In contrast, left ventricular end-diastolic volume (LVEDD) and interventricular septal thickness were comparable between the two groups. Interestingly, the anterior mitral leaflet angle and coaptation depth were greater in the aSMR cohort, whereas the posterior leaflet angle was lower compared with vSMR participants.

### PMR vs. SMR

Participants with PMR had a median age of 70.0 years (IQR 64.0–74.0) and showed a higher proportion of women (55.9%). The prevalence of diabetes mellitus was numerically higher in participants with PMR than in those with SMR (8.1% vs. 4.3%), whereas the prevalence of heart failure and hypertension was comparable between the groups. However, participants with PMR had a significantly higher NYHA functional class (*p* = 0.008). NT-proBNP levels were comparable to those observed in aSMR but lower than in vSMR (167.5 ng/l [IQR 103.0–489.5 ng/l] vs. 143.5 ng/l [85.3–368.0 ng/l] vs. 445.0 ng/l [118.5–960.5 ng/l], respectively, Table 1).

Regarding echocardiographic parameters, severe MR was more prevalent in participants with PMR than in those with SMR (60.3% vs. 38.5%, *p* = 0.003). Participants with PMR showed the highest LVEF (58.4% vs. 57.9% in aSMR vs. 47.4% in vSMR), whereas LVEDD was smaller than in both SMR groups (47.8 mm vs. 48.9 mm in aSMR vs. 49.6 mm in vSMR). Left atrial strain (LAS Global Peak All) was markedly higher in PMR than in the other groups. In contrast, there were no relevant differences in the left atrial volume indexed to body surface area (LASV BSA bp), tricuspid annular plane systolic excursion (TAPSE), or the E/A ratio compared with the SMR groups (Table 2).

**Fig. 5:**
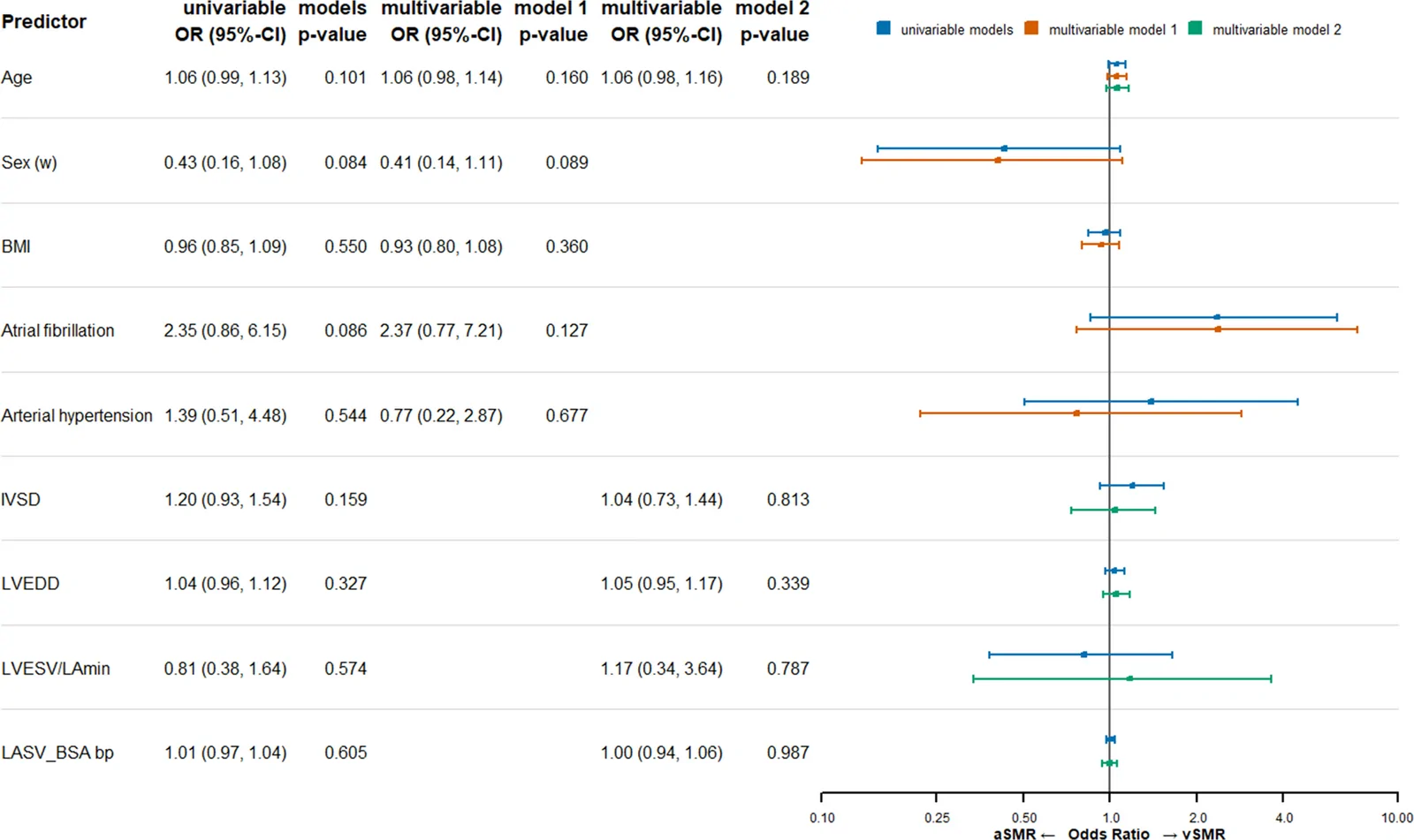
Forest plot showing odds ratios (ORs) and 95% confidence intervals (CIs) from univariable and multivariable logistic regression analyses comparing aSMR with vSMR. (BMI = body mass index, IVSD = interventricular septum enddiastolic, LVEDD = left ventricular enddiastolic diameter, LVESV = left ventricular endsystolic volume, LAmin = minimal left atrial volume, LASV_BSA bp = left atrial endsystolic volume indexed to body surface area biplane, aSMR = atrial secondary mitral regurgitation, vSMR = ventricular secondary mitral regurgitation)

In univariable logistic regression analyses, no statistically significant associations were observed between vSMR and aSMR and comorbidities such as atrial fibrillation and arterial hypertension, nor with echocardiographic parameters including left ventricular end-diastolic diameter or interventricular septal thickness. However, female sex was associated with a more than 50% lower odds of vSMR, whereas atrial fibrillation was associated with more than a twofold higher odd. In addition, arterial hypertension was associated with a 39% increase in the odds of vSMR. Echocardiographic parameters, including intraventricular septum diastolic (IVSd), left ventricular end-diastolic diameter (LVEDD), left ventricular endsystolic volume indexed to minimal left atrial volume (LVESV/LAmin), and left atrial endsystolic volume indexed to body surface area biplane (LASV_BSA), showed substantially weaker associations (Fig. 5).

In the multivariable regression model, atrial fibrillation was the only statistically significant predictor, being associated with a threefold risk of vSMR. Female sex was again associated with a more than 50% lower odds of vSMR, although this effect did not reach statistical significance. Body mass index and arterial hypertension were no relevant predictors in the adjusted model. Similarly, none of the echocardiographic parameters, including IVSd, LVEDD, LVESV/LAmin, and LASV_BSA, were statistically significant or clinically relevant predictors of vSMR in the multivariable analysis (Fig. 5).

**Fig. 6:**
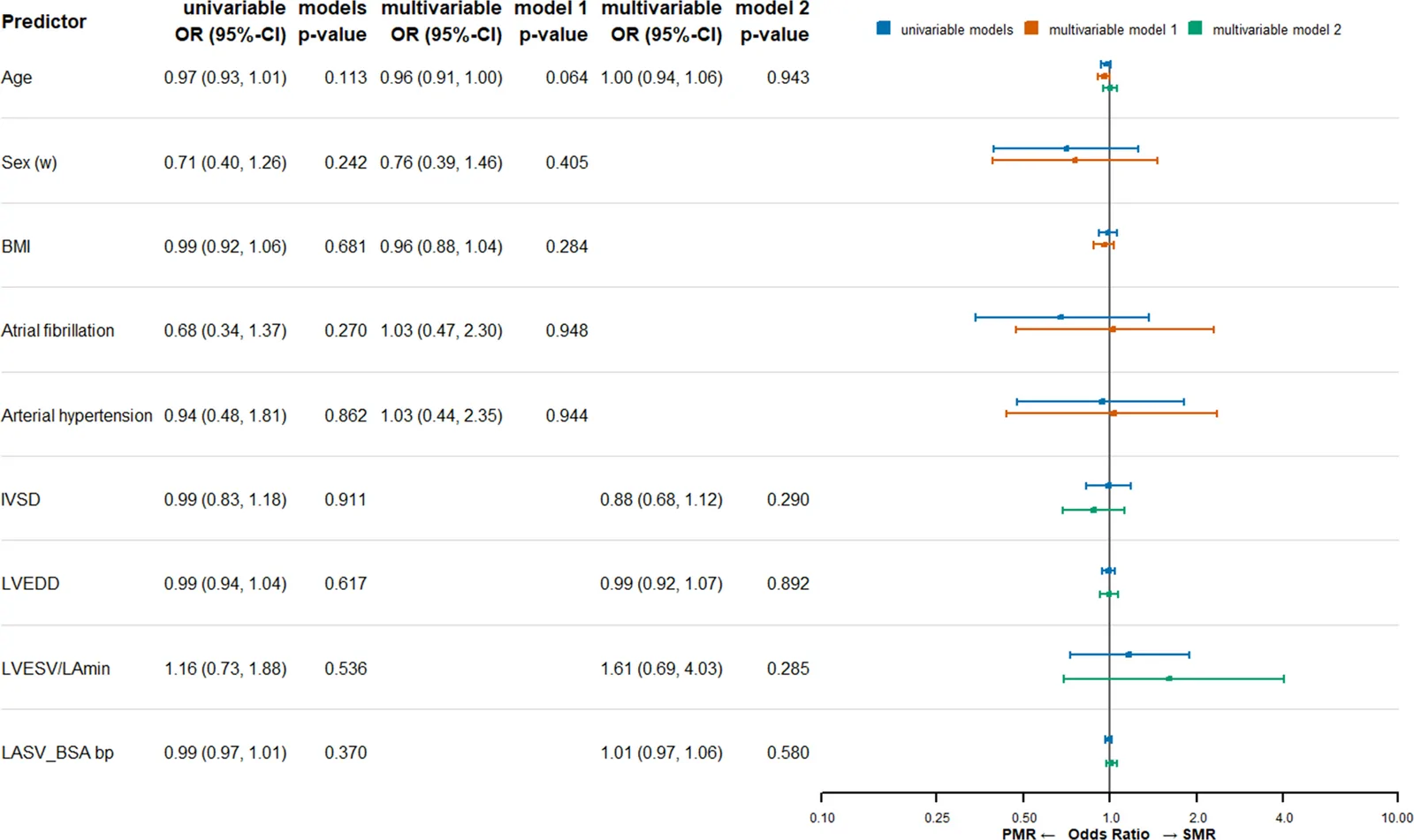
Forest plot showing odds ratios (ORs) and 95% confidence intervals (CIs) from univariable and multivariable logistic regression analyses comparing PMR with SMR. (BMI = body mass index, IVSD = interventricular septum enddiastolic, LVEDD = left ventricular enddiastolic diameter, LVESV = left ventricular endsystolic volume, LAmin = minimal left atrial volume, LASV_BSA bp = left atrial endsystolic volume indexed to body surface area biplane, PMR = primary mitral regurgitation, SMR = secondary mitral regurgitation)

When comparing PMR and SMR, although there were also no statistically significant differences to be found in univariable or multivariable models, participants with PMR tended to be younger. Female sex was associated with numerically lower odds of SMR compared with PMR (OR 0.71, 95% CI 0.40–1.26), although this association did not reach statistical significance. Atrial fibrillation was not significantly associated with MR subtype in either univariable (OR 0.68, 95% CI 0.34–1.37) or multivariable analysis (OR 1.03, 95% CI 0.47–2.30). The numerical association with PMR observed in univariable analysis was attenuated after multivariable adjustment. BMI, arterial hypertension, IVSd, LVEDD, LSESV/Lamin, and LASV/BSA were not significantly associated with MR subtype in either univariable or multivariable analysis (Fig. 6).

### Follow-up

**Fig. 7:**
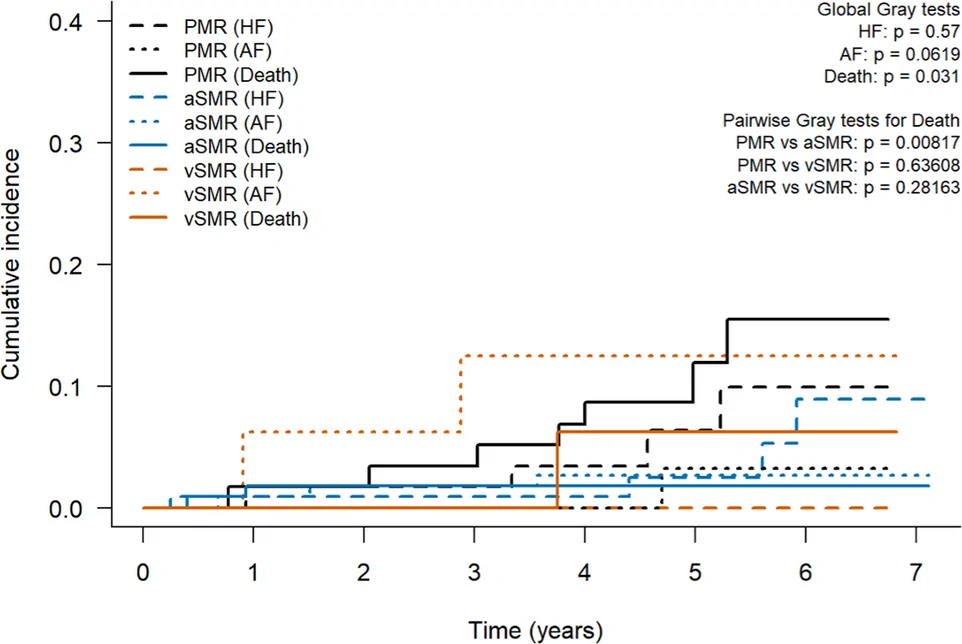
Cumulative incidence analyses of new-onset heart failure and death. (PMR = primary mitral regurgitation, aSMR = atrial secondary mitral regurgitation, vSMR = ventricular secondary mitral regurgitation, HF = heart failure, AF = atrial fibrillation)

Follow-up data were available for 185 participants (58 in PMR, 111 in aSMR and 16 in vSMR). Over a median follow-up of 4.56 years (range 0.39 to 7.11), corresponding to a total of 906.4 patient-years, 7 participants with PMR (288.0 patient-years), 2 with aSMR (545.0 patient-years), and 1 with vSMR (73.4 patient-years) died. Mortality was significantly higher in participants with PMR than in those with aSMR (*p* = 0.0077). In the time-to-first-event analysis, incident heart failure was diagnosed in 4 participants with PMR and 4 with aSMR, while incident atrial fibrillation occurred in 1 participant with PMR, 4 with aSMR, and 2 with vSMR (Fig. 7).

## Discussion

In this population-based cohort of 8543 participants, we identified 216 cases of moderate or severe MR, of which 68.5% were classified as SMR. Among these, aSMR was by far the most prevalent subtype, accounting for 84.5% of SMR cases. MR severity was higher in participants with PMR than in those with SMR.

The prevalence of moderate or severe MR was higher in our cohort than that reported by Dziadzko et al. (2.53% vs. 0.46%) in a retrospective community-based study of 29,390 individuals [11]. However, direct comparison of MR prevalence between our cohorts should be interpreted with caution because of substantial differences in study design and echocardiographic assessment. While Dziadzko et al. applied a comprehensive integrative approach including quantitative measures for MR grading, the HCHS echocardiographic protocol was not specifically designed for the evaluation of valvular heart disease; MR severity was primarily assessed qualitatively [11]. However, all HCHS participants underwent standardized echocardiographic assessment irrespective of clinical indication, allowing for the systematic detection of MR in a population-based cohort. In contrast, Dziadzko et al. calculated community prevalence based on cases identified among residents undergoing clinically indicated echocardiography [11]. Furthermore, Dziadzko et al. included only patients with isolated MR.

In a subcohort of 727 patients from the same community and study period with stored echocardiographic images available for detailed assessment of MR etiology and mechanism, Dziadzko et al. reported a proportion of patients with SMR comparable to that observed in our cohort (65.3% vs. 68.5%) [6]. However, in that study, the majority of patients were classified as having vSMR. This discrepancy is likely attributable to differences in the underlying study populations. In contrast to our population-based cohort, Dziadzko et al. retrospectively included patients with known moderate-to-severe MR who were referred for clinical evaluation [6]. These patients were therefore more likely to be symptomatic and to exhibit higher comorbidity burdens, which is consistent with their reported findings [6]. Moreover, vSMR has been shown to be associated with more pronounced symptoms, irrespective of left ventricular function [12]. This is supported by our observation that individuals with vSMR more frequently reported advanced dyspnea and higher levels of NT-proBNP. In line with our findings, Okamoto et al. reported higher NT-proBNP levels in vSMR in a cohort of 378 patients with SMR [2].

The proportion of PMR was comparable to that reported by Dziadzko et al., accounting for approximately one-third of participants and both regurgitant volume and effective regurgitant orifice area were higher in PMR than in SMR [6]. However, participants in our cohort were older, which is likely attributable to the HCHS inclusion criterion of ≥ 45 years, resulting in the exclusion of younger individuals. The same reason may account for the higher prevalence of atrial fibrillation, diabetes mellitus, and hypertension in our PMR cohort [6].

Furthermore, patients in clinical cohorts, such as that analyzed by Dziadzko et al., may present with greater volume overload, as suggested by the higher LVEDD observed in their cohort [6]. In contrast, the only mildly elevated NT-proBNP levels observed in the present analysis may reflect a relatively clinically stable patient population. When comparing PMR with SMR, female sex showed a numerical tendency toward PMR rather than SMR, although this association did not reach statistical significance. No significant differences in age, BMI, comorbidities, or echocardiographic parameters were observed between MR subtypes.

Consistent with previous studies, we observed a higher proportion of women in the aSMR group, although this difference did not reach statistical significance [2, 6]. It should be noted, however, that this study was not powered to detect subtle demographic differences between SMR subtypes.

The higher all-cause mortality observed in participants with PMR may partly reflect the greater MR severity in this group. In contrast to previous studies reporting the highest excess mortality in vSMR, the small number of outcome events may have contributed to the observed differences in mortality between MR subtypes [6]. Unexpectedly, and in contrast to previous reports, we found that left atrial parameters, such as LASV BSA biplane and the LASV BSA, were comparable between the aSMR and vSMR cohort. Similarly, left ventricular end-diastolic volume and diameter did not differ significantly between the two groups. Moreover, atrial fibrillation was more prevalent among vSMR than among those with aSMR, and a higher proportion of participants with vSMR developed incident atrial fibrillation during follow-up, although the small number of events limits interpretation. These findings challenge the prevailing pathophysiological concept that aSMR is primarily driven by atrial fibrillation in the context of heart failure with preserved ejection fraction (HFpEF) and elevated left atrial pressure [4].

Atrial fibrillation and HFpEF share common risk factors, and data from the Framingham study have identified atrial fibrillation as a predictor for the development of HFpEF, but not HFrEF [13–15]. In a prospective cohort of 635 patients with mild-to-moderate aSMR, the absence of atrial fibrillation was a predictor of SMR regression, whereas the presence of atrial fibrillation did not predict disease progression [16]. Conversely, in a large retrospective study including 31,370 patients, atrial fibrillation was associated with an increased risk of incident aSMR [17]. This is in line with findings from Deferm et al., who reported a reduction in SMR severity following restoration of sinus rhythm in aSMR patients [18].

Interestingly, in our cohort, the E/e′ ratio, a surrogate parameter of diastolic dysfunction, was higher in the vSMR group. This finding contrasts with the results reported by Dziadzko et al., who observed higher E/e′ values in aSMR patients [6]. One possible explanation is that patients with aSMR in our population-based cohort were identified at an earlier disease stage and may develop atrial fibrillation and more pronounced diastolic dysfunction over time. Another explanation is that patients with both atrial fibrillation and SMR may be more symptomatic and therefore more likely to be detected in clinical cohorts than in population-based studies. Furthermore, our population-based cohort comprises clinically stable participants who were not referred for clinical assessment or treatment. As left atrial dimensions are influenced by volume status and may be greater in patients referred for clinical assessment, applying the same cut-off values to our population-based cohort may not be appropriate.

Notably, Dziadzko et al. also reported comparable left atrial volumes between the two SMR subtypes, which is consistent with the present findings [6]. However, we observed a higher left atrial ejection fraction in the aSMR cohort. These findings raise the possibility that current definitions of aSMR, which are inconsistent and largely rely on left ventricular parameters, may not adequately capture the spectrum of atrial remodelling and functional alterations associated with aSMR. These observations raise the possibility that the current definitions of aSMR, largely based on left ventricular parameters, may be insufficient and fail to adequately capture atrial remodelling and dysfunction. Abe et al. defined aSMR as MR associated with left atrial dilatation in the absence of significant left ventricular systolic dysfunction, LV dilatation, or PMR, although universally accepted cut-off values for LVEDD and LVEF are lacking [5]. In contrast, Deferm et al. defined vSMR by the presence of LV dysfunction accompanied by restricted leaflet motion and tethering, whereas aSMR was characterized by preserved LV function and normal leaflet motion [4]. To align our classification with this definition, we incorporated measures of mitral valve geometric distortion, namely tenting area and coaptation depth, into the definition of vSMR.

Dziadzko et al. define vSMR as SMR “with moderate or severe LV remodeling (enlargement or dysfunction or both)” and aSMR as SMR “with no or mild ventricular remodeling but moderate or severe atrial remodeling” [6]. While this approach provides a pathophysiologically meaningful distinction, some patients may not clearly fulfil either set of criteria. For example, the classification may be less straightforward in patients with mildly reduced LVEF and only mild left atrial enlargement but evidence of leaflet tethering. We therefore aimed to facilitate a clear and reproducible classification by applying predefined LVEF cut-off values and incorporating measures of mitral valve geometry, including tenting area and coaptation depth. The LVEF cut-off of 50% is also in line with the current 2025 ESC Guidelines for the management of valvular heart disease [7]. However, a classification system incorporating left atrial function parameters, such as left atrial strain, along with volumetric measures and specific mitral valve geometric indices (e.g., coaptation depth, leaflet angles, and tenting area) may be more pathophysiologically appropriate.

This concept is supported by the mechanistic framework proposed by Deferm et al., who describe leaflet maladaptation due to annular dilatation as the primary mechanism of aSMR, whereas vSMR is driven by increased tethering forces resulting in augmented coaptation depth leaflets angulation[4]. Current classification systems do not explicitly account for these distinct geometric and functional changes. In our cohort, we applied a pragmatic and clinically oriented definition with explicit reference thresholds to enhance reproducibility and comparability. By contrast, Dziadzko et al. defined vSMR based on the presence of moderate-to-severe left ventricular dilatation or dysfunction, while aSMR was defined by moderate-to-severe atrial remodelling [6]. Abe et al. defined aSMR as MR with left atrial dilatation in the absence of significant left ventricular systolic dysfunction, dilatation, or PMR, although no universally accepted cut-off values for LVEDD and LVEF exist [5].

Interestingly, in a small cohort of 52 SMR patients, tenting area was reported to be greater in aSMR patients [19]. Taken together, these inconsistencies highlight the need for prospective studies incorporating advanced echocardiographic imaging, including transesophageal echocardiography and deformation imaging, to better elucidate the distinct pathophysiological mechanisms underlying aSMR and vSMR. Furthermore, longitudinal, prospective studies with serial echocardiographic assessments are needed to elucidate the temporal relationship between atrial fibrillation, atrial remodeling, and the development of aSMR.

Overall, individuals with vSMR in our cohort exhibited a higher burden of comorbidities compared with those with aSMR. This finding contradicts with the observations of Dziazko et al. and Okamoto et al., who investigated the prognostic implications of SMR subtypes in hospital-based cohorts [2, 6]. However, signs of more advanced heart failure in vSMR patients were also reported by Okamoto et al., which aligns with our findings. We hypothesize again that these discrepancies reflect differences in study design: our population-based cohort likely captures earlier stages with generally lower comorbidity rates compared with clinically referred populations. This may also explain the relatively small number of individuals with vSMR observed in our study.

## Limitations

This study has several limitations. First, the relatively small size of the MR subgroups, particularly in the vSMR cohort, and the limited number of outcome events limit statistical power and may have precluded the detection of smaller differences between the groups, thereby limiting definitive conclusions. Accordingly, our findings should be considered hypothesis-generating, and larger studies are warranted to validate these findings.

Second, the exclusion of echocardiographic examinations with insufficient image quality or missing data may have introduced selection bias. Third, although mitral valve assessment was performed independently by two investigators, some degree of misclassification cannot be entirely excluded.

Potential confounding was addressed by applying prespecified multivariable regression models; however, residual confounding remains possible.

Finally, the HCHS echocardiographic protocol was not specifically designed for the assessment of valvular heart disease, and MR severity was therefore primarily evaluated qualitatively.

Notwithstanding these limitations, this is, to our knowledge, the first population-based study specifically investigating PMR, aSMR, and vSMR, thereby providing unique insights into the epidemiology and clinical relevance of these entities in the general population. Furthermore, approximately 900 patient-years of follow-up provide longitudinal data on outcomes in patients with vSMR, aSMR, and PMR in this large cohort.

## Conclusion

In this population-based cohort, the prevalence of moderate or severe mitral regurgitation was 2.53%, of which approximately two-thirds were classified as SMR. When comparing SMR with PMR, PMR was characterized by greater MR severity. While median age and the proportion of women were higher in PMR, comorbidities and echocardiographic characteristics were comparable. All-cause mortality was higher in participants with PMR than in those with aSMR. Among SMR subgroups, aSMR was by far the predominant subtype. Established cardiovascular risk factors were largely comparable between individuals with vSMR and aSMR. However, echocardiographic parameters differed significantly between the two groups, indicating more advanced heart failure and more pronounced left atrial and left ventricular remodelling in the vSMR cohort.

Notably, atrial fibrillation was more prevalent in the vSMR group, challenging current pathophysiological concepts distinguishing aSMR from vSMR in the general population. These findings underscore the need for a more refined classification of SMR subtypes, particularly with regard to their prognostic implications and long-term outcomes, to establish a clinically meaningful and pathophysiologically sound definition of aSMR and vSMR.

## Data Availability

The data underlying this study are not publicly available due to data protection regulations.
